# Arthroscopic evaluation and treatment of a squeaking hip. A case report

**DOI:** 10.1186/s12891-020-03817-x

**Published:** 2020-12-03

**Authors:** Jonathan Bellity, Marc Elkaïm, Didier Hannouche, Rémy Nizard

**Affiliations:** 1 Department of Orthopaedic Surgery, Lariboisière, Hospital, Paris 7 University, Paris, France; 2 Department of Orthopaedic Surgery, Geneva University Hospitals & Faculty of Medicine, Avenue Gabrielle Perret Gentil 4, 1205 Geneva, Switzerland

**Keywords:** Hip, Ceramic, Hip arthroplasty, Squeaking, Arthroscopy

## Abstract

**Background:**

Squeaking of ceramic-on-ceramic total hip arthroplasty is an unexpected complication which occurs in 1- 30% of patients. Revision surgery is required in 0.2% of the cases, when a ceramic fracture is suspected, or in case of severe malposition of the implants, subluxation, or impingement. Hip arthroscopy may be a useful diagnostic and therapeutic option in squeaking hips.

**Case presentation:**

A patient presenting with a pain-free squeaking underwent hip arthroscopy to examine the sliding surfaces and the rim of the acetabulum, and to search for signs of impingement. Thorough lavage and debridement of hip synovitis and fibrous tissue was performed. The squeaking noise immediately disappeared after the surgery. The patient was allowed to fully weight bear as tolerated with 2 crutches for 2 weeks. Two years after the arthroscopy, the patient remained symptom-free.

**Conclusions:**

The potential reasons for hip squeaking in our patient are discussed. Hip arthroscopy may prove useful as a diagnostic and therapeutic option for some patients presenting with a squeaking ceramic-on ceramic hip replacement.

## Background

Due to outstanding tribological properties, the ceramic-on-ceramic (C–C) couple has been accepted as a reliable bearing surface in Total Hip Arthroplasty (THA), especially in young and active patients [1]. However, with the increasing number of C–C prostheses implanted, two complications arose in patients treated with these bearings: fractures of the ceramic and squeaking [2]. Squeaking is a very annoying sound similar to the creaking of a door hinge that occurs during movement of the hip joint. The reported incidence of squeaking varies considerably (from 1 to 30%), and increases in studies where it is enquired specifically. Using the same implant, the incidence of squeaking increased from 3.5% when it was self-reported [3] to 23% when the patients were specifically questioned on noise occurrence [4]. Squeaking is likely to be multifactorial and different hypotheses have been considered to explain this phenomenon, including component positioning, excessive hip range of motion, and larger diameter sizes [4]. It is usually transitory, reproducible only in extreme flexion, and does not influence patients’ satisfaction and outcomes, although patients with non-noisy hips are 1.7 times more likely to report a forgotten joint [5]. Sometimes, it is more permanent and very embarrassing for the patient, occurring also during normal gait. Revision surgery is required in less than 0.2% of the cases [6]; but it is advocated in persisting and painful squeaking, when there is a suspicion of a fracture or chipping of the ceramic liner, severe malposition of the implants, subluxation, and impingement. In contrast, a transitory and painless squeaking may resolve spontaneously in some patients and can be managed with a watch- and see approach, after a thorough clinical and radiological evaluation. However, surveillance is sometimes unacceptable for patients, as squeaking may seriously affect their quality of life and be responsible for harassment, anxiety, sick leaves and social withdrawal [7].

Hip arthroscopy has become a mainstream treatment for a variety of hip disorders, including in the management of the painful hip arthroplasty [8]. We present a documented case of a 53-year-old patient, who underwent hip arthroscopy for a squeaking C–C hip, with a successful mid-term result. To our knowledge, there is no report considering hip arthroscopy in such condition.

## Case presentation

A 53-year-old patient, who had no co-morbidities, underwent in 2003 a C–C THA for advanced primary osteoarthritis on his left hip. At that time, the patient was an airline pilot. His body mass index was 26.8 and remained unchanged during the follow-up period after THA. He used to be a recreational rugby player. The patient was operated through the postero-lateral approach. The femoral stem (Cerafit™) was a straight tapered rough cementless stem (made of TiAl6V4 alloy), fully coated with an 80-micron hydroxyapatite layer, with a 12/14 Morse taper. The press-fit implanted socket (Cerafit™) was hemispherical (50-mm in diameter), coated with an 80-micron thick hydroxyapatite layer and securely fixed with two additional screws. The C–C bearing was made of 3d generation surgical grade alumina ceramic (Ceraver-Osteal™) with high purity, high density, and an average grain size of less than 2 microns. The ceramic liner was fixed inside the metal-back with an inverted Morse-taper cone (slope 5°42′, depth 10 mm), and had an overlip compared to the socket. The ceramic femoral head was 32-mm in diameter with a medium neck length. During surgery, no impingement was noticed between the femoral neck and the socket.

The patient had a good early result. He fully recovered and was able to return to work three months later after a mandatory independent control of the occupational physician. At that time, he had no squeaking and the range of motion was 100° in flexion, 40° in abduction, 20° in adduction, 40° in external rotation, and 10° in internal rotation. He remained symptom-free until May 2011. After a fall from his height, the patient noticed the sudden occurrence of a squeaking, which occurred mainly when he walked and when he kneeled.

**Fig. 1:**
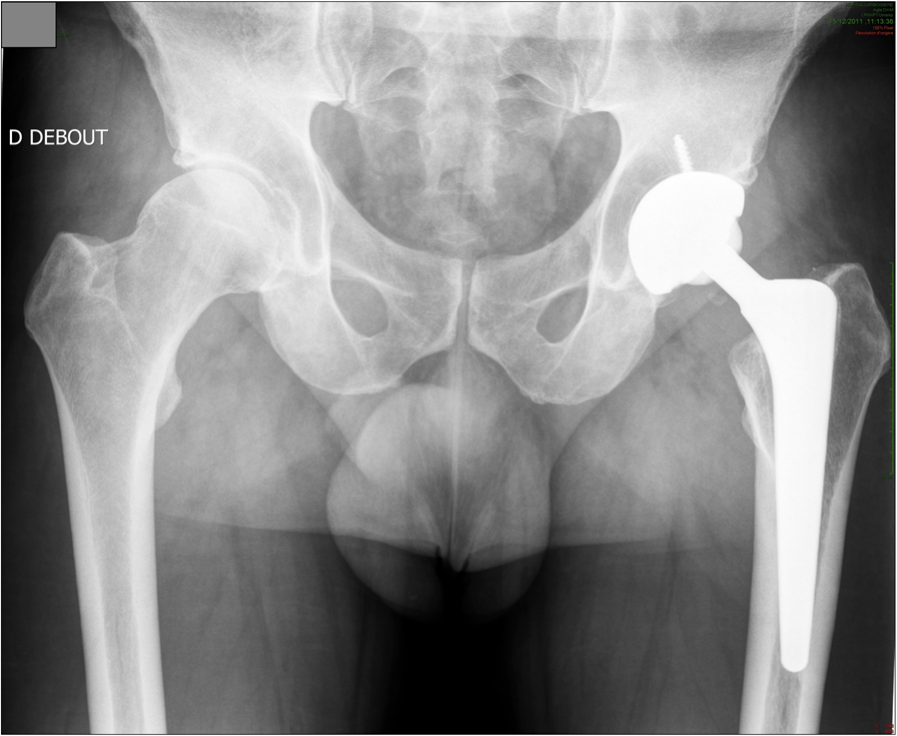
Pelvic post operative X-ray

**Fig. 2:**
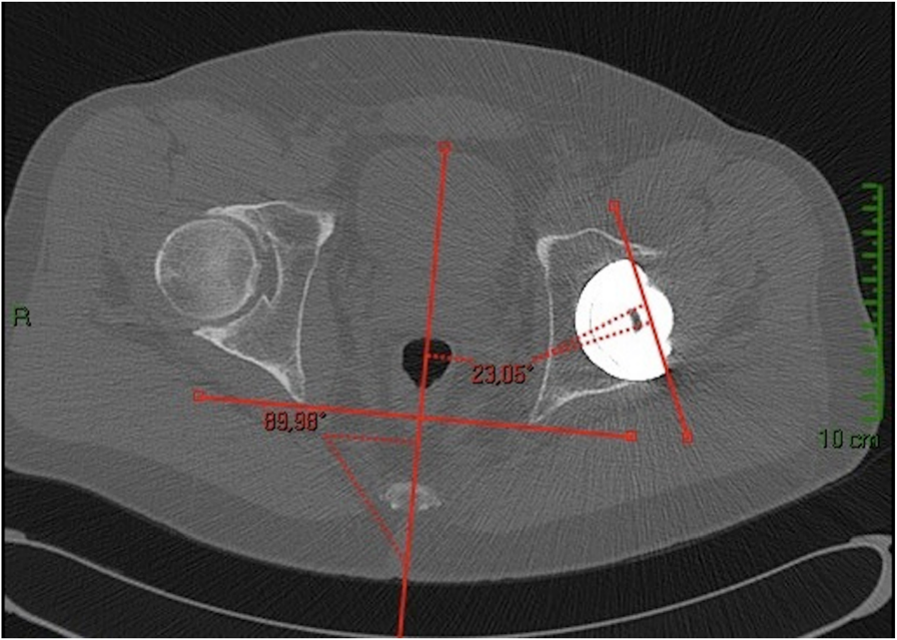
CT-scan showing cup anteversion of 23°

**Fig. 3:**
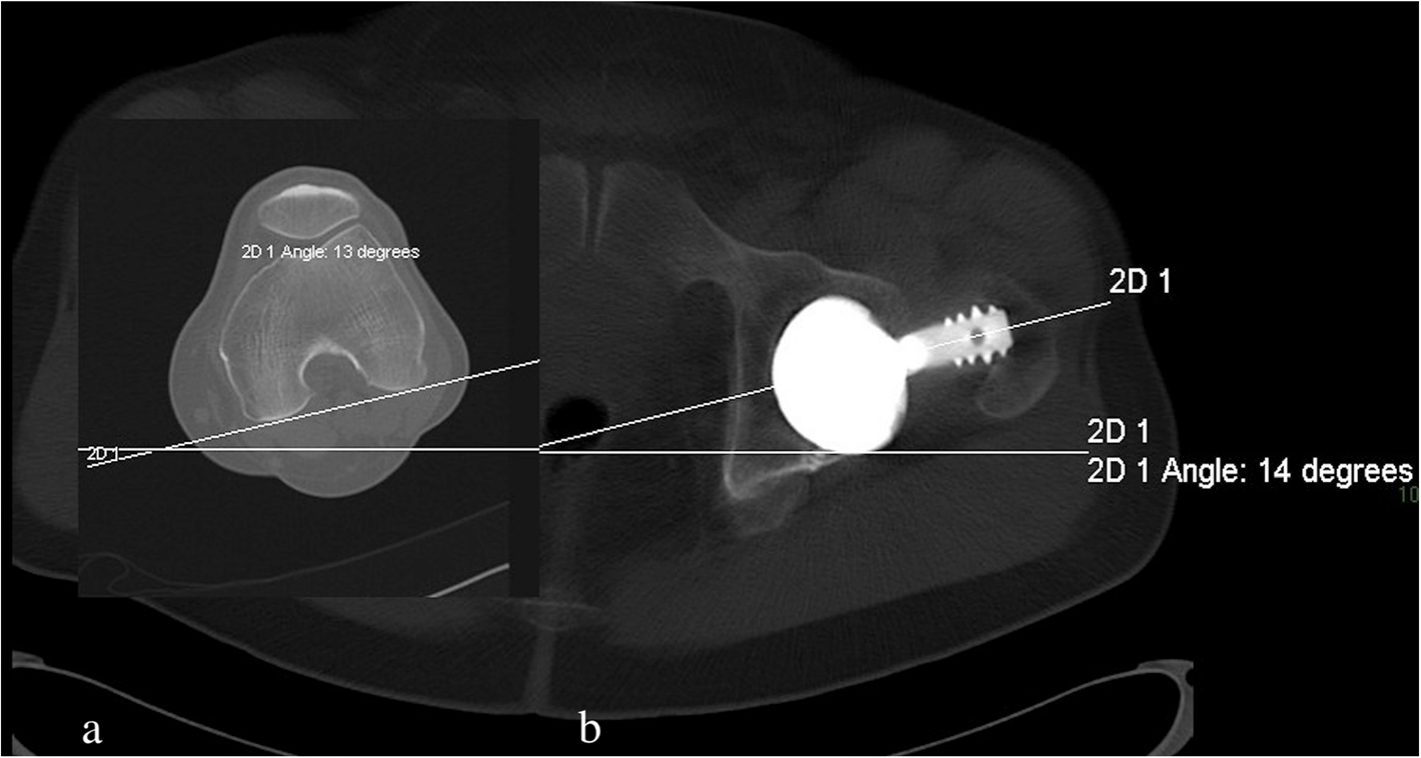
**a** and **b**: CT-scan showing 13° of femoral distal condyle rotation and 14° of stem frontal angle. The stem anteversion (1°) is obtained by subtracting the condylar rotation and the stem angle

On clinical examination, hip range of motion was pain-free and unchanged. Radiographic examination did not show any modification, as compared to previous ones (Fig. 1). A pelvis CT-scan was performed to measure cup and femoral stem orientation, and to detect a potential fracture of the ceramic. The cup inclination angle was 49.6°, cup anteversion was 23° (Fig. 2); the femoral stem was well-aligned with a reduced anteversion of 1° (Fig. 3a and b). The right native hip had 18° of acetabulum anteversion and 18° of femoral neck anteversion. There were no signs of ceramic fracture on both the femoral head and the liner, and no signs of implant loosening.

The patient underwent a hip arthroscopy one week later for inspection of the bearing surfaces, joint debridement and lavage. Full-weight bearing was allowed until the operation. The procedure was performed under general anesthesia, supine on a traction table. Adequate hip distraction was confirmed first using fluoroscopy. A standard anterolateral viewing portal was used with an additional anterior portal. Care was taken to avoid any contact between the bearing surfaces and the instruments that were inserted under fluoroscopy. The hip joint was visualized using a 70°-arthroscope. A transverse capsulotomy was performed in between the two portals using an arthroscopic blade to facilitate the working space in the peripheral compartment after traction release. Examination of the central compartment did not reveal any fracture of the ceramic liner, especially on its rim, and no evidence of stripe wear. The femoral head was also intact, although its entire surface could not be seen despite internal and external rotation maneuvers. There was no evidence of anterior neck-cup impingement. An important synovitis of the hip was visualized at the anterior compartment, and was shaved as well as the remaining fibrous tissue on the superior aspect of the acetabulum. The squeaking noise immediately disappeared after the surgery. The patient was allowed to fully weight bear as tolerated with 2 crutches for 2 weeks. His walking distance was quickly unlimited and so was his biking range. He flew again only a couple of weeks later. Two years after the arthroscopy, the patient remained symptom-free.

## Discussion and conclusion

Squeaking is an unpredictable complication of C–C bearing surfaces, which has been attributed to several factors. It can be related to patient’s body mass index [9], specific designs of the femoral stem [10] or the socket [11, 12], femoral head size (> 36 mm) [13], and short neck length [14]. Also, much emphasis has been put on implant positioning, especially on the socket side. Excessive [15, 16] or insufficient [17] cup anteversion, or a combined femoral and socket anteversion of more than 75° [18] have been associated with squeaking. In our patient, cup inclination and anteversion were in the accepted range of 25 ± 10 degree of anteversion and 45 ± 10 degree of inclination, and the sum of femoral stem and socket anteversion were far below 75°. However, the femoral anteversion was low (1°), as compared to the opposite femur, and to the average 15° anteversion in a normal adult hip. Patel et al. [19] examined the influence of cup and stem orientations on impingement free range of motion of hip implants. The lack of femoral stem anteversion causes the hip to be externally rotated and increases the risk of anterior impingement and posterior subluxation, thus responsible for edge loading and abnormal stripe wear at the posterior aspect of the cup [20].

Recently, several articles have pointed out the role of the functional orientation of the acetabular component on the occurrence of mechanical complications after THA [21]. Tezuka et al. [22] reported that static measurements, which are based on standard supine coronal X-Rays, should be abandoned in favor of a functional safe zone taking into account the specific patient’s spino-pelvic mobility in the sagittal plane, which may modify cup orientation in the standing, sitting and supine positions. An increased posterior pelvic tilt in a standing position thus increases the risk of anterior dislocation, while an increased anterior pelvic tilt in a sitting position increases the risk of posterior dislocation, especially in stiff lumbar spines or after spinal pelvic fusion. Interestingly, in a series of 18 patients with a squeaking hip during deep flexion, Pierrepont et al. [23] showed that the functional orientation of the acetabular component was a good predictor of squeaking and that the mean functional anteversion of the acetabular component in the sitting position was significantly less in the squeaking group than in the control group. In a finite element investigation, the same group later showed that patients with an increased anterior pelvic tilt (thus reducing the functional anteversion of the acetabular component) in the sitting position were more susceptible to posterior edge-loading [24]. The relation between squeaking and edge-loading has been reproduced experimentally by Taylor et al. [25], who showed that squeaking systematically occurred after the onset of stripe wear and was due to a combined phenomenon of edge-loading and lack of joint lubrication.

Another explanation for the occurrence of squeaking in our patient could be the presence of a microcrack and the release of small ceramic grains during the trauma, which were entrapped into the joint. In a three-dimensional model of in vitro hip kinematics, a 500 microns-microseparation during the swing phase of walking did not replicate squeaking, whereas entrapment of third-body ceramic particles between the sliding surfaces could generate clinically relevant noises [26]. Recently, in a pin-disc testing of a C–C coupling, Fukui et al. [27] showed that squeaking was related to the presence of microcracks at the surface of the worn ceramic, and not to an extra-articular impingement of the femoral neck.

Finally, squeaking could be related to a fracture of the ceramic liner. Abdel et al. [28] and Dacheux et al. [29] reported on patients who had a painful squeaking due to unnoticed ceramic liner fractures. Standard X-Rays are insufficient to diagnose this type of fractures, which can be detected with a dual energy CT-scan [30, 31] or with synovial fluid analysis to quantify the presence of ceramic debris [32]. The relation between squeaking and ceramic fracture is, however, controversial. In a series of 100 patients, 5% of the patients had a squeaking, but the authors stated that this was an isolated phenomenon without any consequence at 10 years, and that there was no relation between squeaking and ceramic fracture [16]. It seems plausible that squeaking is more related to ceramic wear, as shown recently by Baruffaldi et al. [33]. The authors recorded 46 patients who had various noises from their joint, with a sensibility range from 20 Hz to 20 kHz. They showed that high frequency noises such as squeaking are audible years before the indication for revision, and are an indicator for a significant ceramic wear in progress.

Considering the above experimental data, and the possible release of ceramic grains in our patient, we made the hypothesis that hip arthroscopy could be an option for both diagnostic and therapeutic reasons. Before the arthroscopy, he had a CT-scan to search for a ceramic fracture and possible signs of impingement on the femoral neck. The goal of the arthroscopy was to further explore the sliding surfaces, to examine to femoral neck and the rim of the acetabulum, and to perform a joint debridement with lavage to evacuate third-body particles that might be incarcerated. We believe that arthroscopy might be an interesting option in case of transitory and pain-free squeaking, especially in young and active patients, who report a significant psychological and social impact. In case of painful squeaking, and when a fracture is suspected, revision surgery must be strongly recommended to exchange the implants.

In conclusion, hip arthroscopy may prove useful as a diagnostic and therapeutic option for some patients presenting with a squeaking C–C hip replacement. More data from different surgeons using different prostheses designs have to be gathered to better define its limits.

## Data Availability

The datasets used and/or analyzed during the current study are available from the corresponding author on reasonable request.

## Abbreviations

C–C: Ceramic-on-Ceramic
THA: Total Hip Arthroplasty
CT-Scan: Computerized Tomography-Scan
